# What is slough? Defining the proteomic and microbial composition of slough and its implications for wound healing

**DOI:** 10.1111/wrr.13170

**Published:** 2024-04-01

**Authors:** Elizabeth C. Townsend, J. Z. Alex Cheong, Michael Radzietza, Blaine Fritz, Matthew Malone, Thomas Bjarnsholt, Karen Ousey, Terry Swanson, Gregory Schultz, Angela L. F. Gibson, Lindsay R. Kalan

**Affiliations:** ^1^ Department of Medical Microbiology and Immunology University of Wisconsin School of Medicine and Public Health Madison Wisconsin USA; ^2^ Microbiology Doctoral Training Program University of Wisconsin‐Madison Madison Wisconsin USA; ^3^ Medical Scientist Training Program University of Wisconsin School of Medicine and Public Health Madison Wisconsin USA; ^4^ Infectious Diseases and Microbiology Western Sydney University Sydney Australia; ^5^ Department of Immunology and Microbiology University of Copenhagen Copenhagen Denmark; ^6^ Department of Clinical Microbiology Copenhagen University Hospital Copenhagen Denmark; ^7^ International Wound Infection Institute London UK; ^8^ Institute of Skin Integrity and Infection Prevention University of Huddersfield West Yorkshire UK; ^9^ Department of Obstetrics and Gynecology University of Florida Gainesville Florida USA; ^10^ Department of Surgery University of Wisconsin School of Medicine and Public Health Madison Wisconsin USA; ^11^ Department of Biochemistry and Biomedical Sciences McMaster University Hamilton Ontario Canada; ^12^ M.G. DeGroote Institute for Infectious Disease Research McMaster University Hamilton Ontario Canada; ^13^ David Braley Centre for Antibiotic Discovery McMaster University Hamilton Ontario Canada

**Keywords:** biofilm, chronic wounds, microbiome, proteomics, slough

## Abstract

Slough is a well‐known feature of non‐healing wounds. This pilot study aims to determine the proteomic and microbiologic components of slough as well as interrogate the associations between wound slough components and wound healing. Ten subjects with slow‐to‐heal wounds and visible slough were enrolled. Aetiologies included venous stasis ulcers, post‐surgical site infections and pressure ulcers. Patient co‐morbidities and wound healing outcome at 3‐months post‐sample collection was recorded. Debrided slough was analysed microscopically, through untargeted proteomics, and high‐throughput bacterial 16S‐ribosomal gene sequencing. Microscopic imaging revealed wound slough to be amorphous in structure and highly variable. 16S‐profiling found slough microbial communities to associate with wound aetiology and location on the body. Across all subjects, slough largely consisted of proteins involved in skin structure and formation, blood‐clot formation and immune processes. To predict variables associated with wound healing, protein, microbial and clinical datasets were integrated into a supervised discriminant analysis. This analysis revealed that healing wounds were enriched for proteins involved in skin barrier development and negative regulation of immune responses. While wounds that deteriorated over time started off with a higher baseline Bates‐Jensen Wound Assessment Score and were enriched for anaerobic bacterial taxa and chronic inflammatory proteins. To our knowledge, this is the first study to integrate clinical, microbiome, and proteomic data to systematically characterise wound slough and integrate it into a single assessment to predict wound healing outcome. Collectively, our findings underscore how slough components can help identify wounds at risk of continued impaired healing and serves as an underutilised biomarker.

## 1 INTRODUCTION

Chronic, non‐healing wounds impose a significant, underappreciated burden to affected individuals and the healthcare system. An estimated 2%–10% of the general population in Australia, the United Kingdom and the United States suffer from chronic wounds.^1^, ^2^, ^3^, ^4^ Individuals with conditions known to impair wound healing, such as peripheral arterial disease, venous insufficiency, immune‐compromised, obesity, diabetes, impaired sensation, and spinal cord injuries are at the highest risk for developing chronic wounds.^5^ With the prevalence of these comorbidities on the rise, chronic wounds are anticipated to pose a growing burden for patients and the healthcare system.^2^, ^3^ Thus, identifying biomarkers to distinguish chronic wounds that are likely to heal from those that may benefit from intensive therapies to promote healing is a critical imperative.

A hallmark feature of chronic wounds is the presence of slough, which mainly consists of devitalized tissue that overlays the wound bed. Slough is hypothesized to arise as a byproduct of prolonged wound inflammation.^6^, ^7^, ^8^ On a macro level, slough has highly variable physical characteristics ranging in consistency, colour, odour, and attachment to the wound bed even across a single wound's surface.^6^, ^8^ Subsequently, the appearance varies widely wound‐to‐wound and patient‐to‐patient. Although, to date, there are no studies interrogating slough directly, assessments of exudative fluid from surface of chronic wounds and wound biopsies suggest that the wound surface and associated slough is enriched for various types of collagen, extracellular matrix proteins, matrix metalloproteases, and proteins related to inflammatory immune responses.^9^, ^10^, ^11^, ^12^, ^13^, ^14^, ^15^ Slough can also be infiltrated by an array of bacterial either as single cells or by forming aggregates and biofilm.^16^, ^17^, ^18^, ^19^, ^20^, ^21^ However, due to the highly variable appearance and inconsistencies in even defining slough between providers, it is difficult to distinguish slough with or without microbial biofilm from infected wound exudate.^16^, ^22^ Ultimately, slough's variable nature has led to inconsistent clinical approaches to wound management.

One dominant theory proposes that slough inhibits wound healing by prolonging the inflammatory phase of healing, preventing the formation of granulation tissue and subsequent wound contraction. Slough is commonly associated with biofilm, although limited evidence exists to support the idea that slough is primarily microbial in nature. Slough may serve as a reservoir attractive to bacteria on the wound bed that subsequently promotes biofilm formation, however this is also challenging to quantify.^6^ In the absence of conclusive data, standard chronic wound care focuses on proper debridement to remove devitalized tissue, reduce potential surface microbial burden, and ideally return the wound to an acute state to stimulate tissue repair.^23^ However, <50% of wounds respond or go on to heal following debridement.^24^ Conversely, some wounds with slough present will heal without debridement, suggesting that the presence of slough does not always indicate that healing is disrupted.^8^

Despite it being a common feature of chronic wounds, a detailed molecular characterisation of the host and microbial components within slough from different wound aetiologies is missing. A systematic analysis of slough composition and factors associated with wound healing outcomes could shape wound treatment strategies and aide in triaging high risk patients into specialty care.

With this pilot study, we aim to characterise the human and microbial components of slough collected from wounds of various aetiologies. Collectively we show that wound slough is primarily composed of proteins associated with the structure and formation of the skin, blood clot formation, and various immune responses. Wound slough is highly polymicrobial and exhibits signatures associated with both wound aetiology and location on the body. Finally, slough protein profiles from wounds with a healing trajectory are significantly different than slough protein profiles from non‐healing wounds, suggesting they may serve as a prognostic marker. Rather than being discarded, slough may be a critical indicator to predict if a wound is more likely on the trajectory toward healing or at risk of deteriorating.

## 2 METHODS

### 2.1 Subject identification and enrolment

Adults 18 years or older with chronic wounds were recruited from UW‐Health Wound Care Clinics under an IRB approved protocol (Study ID: 2020‐1002). Examples of wounds identified for possible inclusion included and were not limited to, chronic or non‐healing diabetic ulcers, pressure ulcers, venous ulcers, surgical or procedural wounds, trauma wounds, burn wounds, and wounds of unknown or other aetiology. On the day of sample collection, subject wounds were measured, evaluated and scored according to the Bates‐Jensen Wound Assessment Tool.^25^ Information related to the wound's aetiology and care, wound measurements from the most recent previous visit, and patient co‐morbidities were extracted from the medical record. Digital photos of the wound were taken before and after the debridement procedure. Swabs for microbiome analysis of the wound edge and centre were taken prior to washing the wounds and collected using Levine's technique and placed into 300 μl of DNA/RNA Shield (Zymo Research, Irvine, CA) and stored at −80°C until further processing. Swabs were spun down using DNA IQ Spin Baskets (Promega, Madison, WI) and DNA was extracted. Swabs designated for microbial culture were taken from the wound centre using Levine's technique into 1 ml of liquid Amies (Copan Diagnostics Inc., Murrieta, CA). Swabs were stored at 4°C for <2 h before being processed for microbial culture.

All subjects received sharp debridement of their wounds. Prior to debridement wounds were washed with soap and rinsed with water. Debridement was performed by a skilled practitioner with surgical instruments such as scalpel, curette, scissors, rongeur, and/ or forceps. Removed slough material was collected into 1 ml of DNA/RNA Shield (Zymo Research, Irvine, CA) and stored at 4°C before sectioning for scanning electron microscopy (SEM), fluorescence in situ hybridization (FISH) and proteomics. Remaining slough material was stored at −80°C.

Samples from South Western Sydney Hospital were collected and processed as described by Malone et al.^26^ Adults 18 years or older presenting with a diabetes‐related foot ulcer with visible signs of slough were recruited for the study. The collection of samples and their corresponding patients was undertaken as a sub‐analysis of a larger clinical study, with samples being obtained following written consent. Ethics approval for the larger clinical study and the slough sub‐analysis was approved by South Western Sydney LHD Research and Ethics Committee. All DFUs were debrided and rinsed with 0.9% NaCl prior to specimen collection. For DNA sequencing, patient wound slough was removed from the ulcer base with a dermal curette and immediately stored in RNA Shield (Zymo Research, Irvine, CA) at 4°C for 24 h before being frozen at −80°C until further processing. For PNA‐FISH, tissue specimens were obtained through a dermal ring curette from the wound bed. Following removal, tissue specimens were rinsed vigorously in a phosphate buffer solution (PBS) bath to remove any coagulated blood and to reduce the number of planktonic microorganisms. Tissue specimens were immediately fixed in 4% paraformaldehyde overnight at 4°C, then transferred into 70% ethanol and stored at −20°C.

### 2.2 Microbial culture and bacterial isolate identification

Swabs designated for microbial culture were spun down using DNA IQ Spin Baskets (Promega, Madison, WI). A portion of each sample was serially diluted with 1X phosphate buffered saline and plated onto Tryptic Soy Agar (TSA) with 5% sheep blood (BBL, Sparks, MD) for quantitative bacterial culture. Plates were incubated at 35°C overnight. To isolate culturable bacteria, colonies with distinct morphology were isolated and incubated at 35°C overnight on TSA with 5% sheep blood then single colonies were inoculated into liquid Tryptic Soy Broth (TSB) for overnight incubation. To identify each bacterial isolate, a portion of the overnight TSB culture underwent DNA extraction and sanger sequencing (Functional Biosciences, Madison, WI) of the bacterial 16S ribosomal RNA gene. The remaining portion of the isolate culture was stored in glycerol at −80°C.

### 2.3 DNA/RNA extraction, library construction and sequencing

DNA extraction on samples collected in the USA was performed as previously described with minor modifications.^27^ Briefly, 300 μl of yeast cell lysis solution (from Epicentre MasterPure Yeast DNA Purification kit), 0.3 μl of 31,500 U/μl ReadyLyse Lysozyme solution (Epicentre, Lucigen, Middleton, WI), 5 μl of 1 mg/ml mutanolysin (M9901, Sigma‐Aldrich, St. Louis, MO) and 1.5 μl of 5 mg/ml lysostaphin (L7386, Sigma‐Aldrich, St. Louis, MO) was added to 150 μl of swab liquid before incubation for 1 h at 37°C with shaking. Samples were transferred to a tube with 0.5 mm glass beads (Qiagen, Germantown, MA) and bead beat for 10 min at maximum speed followed by a 30 min incubation at 65°C with shaking, 5 min incubation on ice. The sample was spun down at 10,000 rcf for 1 min and the supernatant was added to 150 μl of protein precipitation reagent (Epicentre, Lucigen, Middleton, WI). Remaining steps followed the recommended PureLink Genomic DNA Mini Kit (Invitrogen, Waltham, MA) protocol for DNA extraction and purification. 16S rRNA gene amplicon libraries targeting the V1–V3 or V4 region were constructed using a dual‐indexing method and sequenced on a MiSeq with a 2×300 bp run format (Illumina, San Diego, CA). Reagent‐only negative controls were carried through the DNA extraction and sequencing process.

Swabs obtained from DFUs in Australia were defrosted on ice prior to DNA extraction. Genomic DNA was extracted using Qiagen DNeasy PowerBiofilm kit (Cat No./ID: 24000‐50) following the manufacturer's instructions. Preparation of the16S library and DNA sequencing was carried out by a commercial laboratory (Ramaciotti Centre for Genomics, University of New South Wales, Australia) on the Illumina MiSeq platform (2×300bp) targeting the V1‐V3 (27f/519r) 16S region.

### 2.4 Sequence analysis

The QIIME2^28^ environment was used to process DNA‐based 16S rRNA gene amplicon data. Paired end reads were trimmed, quality filtered and merged into amplicon sequence variants (ASVs) using DADA2. Taxonomy was assigned to ASVs using a naive Bayes classifier pre‐trained on full length 16S rRNA gene 99% operational taxonomic unit (OTU) reference sequences from the Greengenes database (version 13_8). Using the qiime2R package, data was imported into RStudio (version 1.4.1106) running R (version 4.1.0) for further analysis using the phyloseq package.^29^ Negative DNA extraction and sequencing controls were evaluated based on absolute read count and ASV distribution in true patient samples. Abundances were normalised proportionally to total reads per sample. Data was imported into RStudio running R (version 4.2.1) for analysis. Relative abundance plots were produced using the package ggplot2, where taxa below 1% relative abundance were pooled into an “Other” category. To evaluate the similarity of wound sample microbial community compositions, samples were first rarified to a read depth of 800 and evaluated with the Bray‐Curtis beta diversity. Associations between microbial community composition and wound features were assessed with univariate permutational multivariate analysis of variance (PERMANOVA). Each PERMANOVA was run considering the marginal effects of terms with 9999 permutations using the Adonis2 in the vegan r package.^30^ Spearman correlations were used to evaluate association of bacterial ASV abundance with a points position on the NDMS ordination.

### 2.5 Proteomics

Debrided slough tissue samples were weighed and placed in PowerBead tubes containing 1.4 mm ceramic beads (Qiagen, Germantown, MA) for tissue homogenization, proteomic processing and analysis at the University of Wisconsin Mass Spectrometry and Proteomics Core Facility. In brief, samples were labelled and pooled for multiplex relative mass spectrometry (MS) quantification with the TMTpro 16plex labeling kit (ThermoFisher Scientific, Waltham, MA) and underwent Liquid Chromatography with tandem mass spectrometry on an Orbitrap Elite mass spectrometer (ThermoFisher Scientific). Protein sequences were matched to known human and bacterial proteins, with *Staphylococcus aureus* and *Pseudomonas aeruginosa* serving as representative wound associated gram‐positive and gram‐negative bacteria. Functions associated with each UniProt protein were gathered from the Gene Ontology (GO) database, KEGG Pathways, Reactome Pathways and WikiPathways databases. Data was imported into RStudio for analysis. To determine the most enriched proteins and their associated biologic processes within slough, abundances were normalised proportionally to total abundance per sample and the ranked dataset was analysed via the Gene Ontology enRIchment analysis (GORILA).^31^ This analysis both compared proteins at the top of the list to those at the bottom of the list as well as compared them to general abundances within homo sapiens tissues using minimum‐Hypergeometric statistics. This generated a table of GO Terms, their enrichment in the dataset and False Discovery Rate (FDR) corrected q‐value calculated by the Benjamini–Hochberg procedure. These results were input into the rrvgo^32^ r package for visualisation of significantly enriched GO Terms both via PCoA ordination and Treemap plots of the GO Term hierarchical clusters. Differential protein expression between subject groups was assessed via DEqMS.^33^ Proteins were considered to be differentially abundant in a given group if they displayed log_2_(fold change) >1 and Limma *p* value <0.01. To determine the key biologic processes for smaller sets of proteins, such as those enriched within subject groups or the proteins within each of the k‐means protein clusters (see *Integration of Biologic Data Sets* below), small sets of proteins were submitted as unranked lists to the GO Enrichment Analysis tool.^34^, ^35^ This compared the number of proteins/genes submitted in a particular annotation data category to the expected number in that category for the reference database (homo sapiens). This calculated the fold enrichment, raw Fisher's exact *p* value and FDR *q*‐value for each of the relevant GO Terms. Results were input into rrvgo^32^ for visualisation.

### 2.6 Fluorescence in situ hybridization (FISH)

Formalin‐fixed paraffin‐embedded (FFPE) histological sections were deparaffinised in xylene and rehydrated in a series of ethanol washes (100%, 99%, 95% and 0%). Subsequently, the samples were allowed to hybridise at 46°C for 4 h in hybridization solution (900 mM NaCl, 20 mM Tris pH 7.5, 0.01% SDS, 20% formamide, 2 μM FISH probe). The FISH probe used was a DNA oligonucleotide (EUB388 sequence) with a 3′‐conjugated TEX615 fluorophore (Integrated DNA Technologies, Coralville, IA, USA). Samples were washed in excess wash buffer (215 mM NaCl, 20 mM Tris pH 7.5, 5 mM EDTA) at 48°C for 15 min, dipped into ice cold water, 100% ethanol, drained and air‐dried. Slides were mounted with Prolong Glass antifade mounting medium with NucBlue counterstain (Thermo Fisher Scientific, Waltham, MA) and a glass coverslip of #1.5 thickness and stored flat to cure overnight in the dark. Micrographs were acquired using a Zeiss 780 confocal laser scanning microscope on the red TEX615, blue Hoescht and green GFP (tissue autofluorescence) channels using ×5 and ×63 objectives. Zeiss Zen software was used to analyse tiled images, z‐stacks and generate maximum intensity projections.

### 2.7 PNA‐FISH

As described by Nadler et al.,^36^ formalin‐fixed, paraffin‐embedded samples were cut, deparaffinised and rehydrated following standard procedures. Subsequently, the samples were stained with a PNA‐FISH‐TexasRed‐5‐conjugated universal bacterial (BacUni) 16s rRNA probe (AdvanDx, Woburn, MA, US), incubated and then counterstained with 3 μM 4′,6‐diamidino‐2‐phenylindole (DAPI) (life Technologies, Eugene, OR, USA). The samples were afterward mounted (ProLong Gold Antifade Mountant, Life Technologies) and a coverslip was added (Marienfield, Lauda‐Königshoffen, Germany). Slides were evaluated using a CLSM (Axio Imager.Z2, LSM880 CLSM; Zeiss, Jena, Germany). Images were taken using 405 nm (DAPI) and 561 nm (TexasRed‐5) lasers, as well as a 488 nm laser for visualising the green autofluorescence of the surrounding tissue. Images were subsequently processed with IMARIS 9.2 (Bitplane, Zurich, Switzerland) and presented using “Easy 3D.”

### 2.8 Scanning electron microscopy

Wound slough specimens were rinsed with PBS and fixed overnight in 5 ml of 1.5% glutaraldehyde in 0.1 M sodium phosphate buffer (pH 7.2) at 4°C. Samples were rinsed, treated with 1% osmium tetroxide for 1 h and then washed again in buffer. Samples were dehydrated through a series of ethanol washes (30%–100%) followed by critical point drying (14 exchanges on low speed) and were subsequently mounted on aluminium stubs with a carbon adhesive tab and carbon paint. Samples were left to dry in a desiccator overnight. Following sputter coating with platinum to a thickness of 20 nm, samples were imaged in a scanning electron microscope (Zeiss LEO 1530‐VP) at 3 kV.

### 2.9 Integration of biologic data sets

To reduce the complexity of the proteomics data for integrative analysis protein abundances were normalised, mean centred and grouped via k‐means clustering. The optimal number of protein clusters was determined by the Gap‐Statistics method. Since there was no significant difference in the microbial community composition at the wound edge or centre, as assessed by Bray‐Curtis dissimilarly metric (“Sequence analysis” section above), taxa relative abundances from the wound edge and centre were averaged to create a summative wound slough microbiome for each subject. The 15 microbial ASVs with >1% relative abundance in at least two summative subject slough microbiome samples were included for this analysis. To predict the variables associated with wound healing, the protein cluster, summative slough microbiome and the numerical Bates‐Jensen Wound Assessment score datasets were integrated into a supervised Partial Least Squares—Discriminant Analysis (PLS‐DA, aka. Data Integration Analysis for Biomarker discovery using Latent variable approaches for Omics studies [DIABLO]) via the via MixOmics^37^ R‐studio package.

### 2.10 Statistical analyses

Statistical analyses were conducted in R studio running R (version 4.2.1). Bates Jensen wound assessment scores were analysed via Prism (version 9.2.0).

3

## 4 RESULTS

### 4.1 Subject and wound characteristics

Ten subjects recruited in the United States with wounds of various aetiologies were included in this study (Tables 1 and S1). This is the main subject cohort evaluated. Data from an additional cohort of 13 patients recruited Australia for slough imaging and microbiome assessment is available in Data S1. For the 10 United States patients, prior to sample collection wounds were measured, evaluated and scored according to the Bates‐Jensen Wound Assessment Tool (Tables 2 and S1).^38^ Overall Bates‐Jensen Wound Assessment scores ranged from 26 to 46 (mean 37.4) out of 60 points with higher scores indicating greater wound degeneration. Photos of the wounds before sharp debridement of the superficial wound slough are in Figure S1.

**TABLE 1:** Subject and wound characteristics.

	All subjects (*n* = 10)
Age (yr: Mean ± SD)	66 ± 11
Biologic Sex (M:F)	4:6
Race	
White	9
Black/African American	1
Wound characteristics	
Wound age (yr: Mean ± SD)	2.4 ± 4.5
Wound aetiology	
Pressure ulcer	2
Surgical infection	2
Trauma	2
Venous stasis ulcer	4
Wound location	
Coccyx	2
Shin	4
Posterior lower leg	1
Ankle	3
History of the wound	
Previously debrided	6
Previously infected	5
Current infection	1
Wound outcome at 12 weeks	
Healed	3
Ongoing, stable	4
Deteriorated	3
Patient comorbidities	
BMI (Mean ± SD)	43.0 ± 18.6
Anaemia	2
Heart disease	3
Pre‐diabetes	1
Diabetes	4
Hypertension	5
Lymphedema	2
Neuropathy	1
Paraplegia	2
Peripheral vascular disease	4
Former or current smoker	4
History of alcohol use disorder	1

**TABLE 2:** Bates‐Jensen wound assessment scores by wound healing outcome at 3 months.

	Healed (*n* = 3)	Ongoing (*n* = 4)	Deteriorated (*n* = 3)	All subjects (*n* = 10)
Size	2.00 ± 1.00	3.25 ± 1.71	3.00 ± 2.00	2.80 ± 1.55
Depth	2.67 ± 0.58	2.75 ± 0.5	4.00 ± 1.00	3.10 ± 0.88
Edges	2.67 ± 1.15	3.25 ± 0.5	3.67 ± 0.58	3.20 ± 0.79
Undermining	2.33 ± 2.31	1.50 ± 1.00	2.33 ± 2.31	2.00 ± 1.70
Necrotic tissue type	2.67 ± 0.58	3.00 ± 0.82	3.00 ± 1.00	2.90 ± 0.73
Necrotic tissue amount	2.67 ± 0.58	2.75 ± 0.5	2.67 ± 2.08	2.70 ± 1.06
Exudate type	2.67 ± 0.58	3.25 ± 0.5	3.67 ± 1.15	3.20 ± 0.79
Exudate amount	3.33 ± 0.58	3.50 ± 0.58	4.33 ± 0.58	3.70 ± 1.32
Skin colour surrounding wound	1.00 ± 0	1.75 ± 1.50	2.67 ± 1.53	1.80 ± 1.32
Edema	1.67 ± 1.15	2.50 ± 1.73	2.33 ± 1.53	2.20 ± 1.40
Induration	1.00 ± 0	1.75 ± 0.96	2.00 ± 1.73	1.60 ± 1.08
Granulation	2.00 ± 1.00	3.75 ± 0.50	4.00 ± 0	3.30 ± 1.06
Epithelialization	5.00 ± 0	4.75 ± 0.50	5.00 ± 0	4.90 ± 0.32
Total score	31.67 ± 4.93	37.75 ± 3.86	42.67 ± 3.06	37.4 ± 5.7

*Note*: Data represented as mean ± SD.

Wound status at 3 months post‐sample collection was recorded (Table 1). At this time, three of the subjects' wounds healed, four were ongoing yet stable in size and clinical assessment, and three wounds had deteriorated (e.g., significantly increased in size, depth and/or continued antibiotic resistant infection). The total Bates‐Jensen Wound Assessment Score, and several of the sub‐scores trended higher in wounds that deteriorated compared to those that went on to heal (*p* values <0.1, yet >0.05, Mann–Whitney *t*‐test Table 2). However, none of these comparisons reached statistical significance, likely due to the relatively small number of subjects within each group.

### 4.2 Slough protein composition is associated with wound age and healing trajectory

**FIGURE 1:**
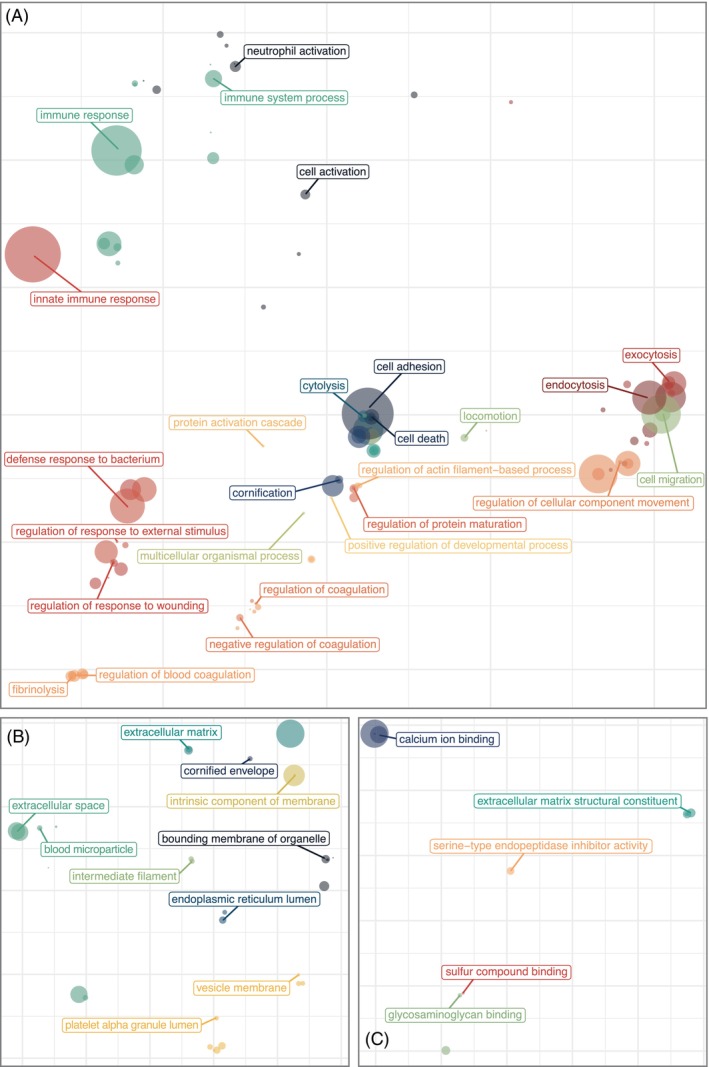
Wound slough is enriched for proteins involved in skin barrier formation, wound healing, blood clotting and various immune functions including responding to bacteria. Debrided slough tissue was sent for proteomic characterisation via mass spectrometry. The most abundant proteins across all samples were input as a ranked list to the Gene Ontology enRIchment analysis (GORILA).^31^ Results were visualised with rrvgo.^32^ For each PCoA plot, distances between points represent the similarity between GO Terms and the size of each point indicates the degree of significance for that terms enrichment in the dataset (the ‐log_10_[FDR q‐value]). (A) PCoA ordination of significantly enriched GO Terms from the biological process ontology. (B) PCoA ordination of GO Terms from the cellular components ontology. (C) PCoA ordination of significantly enriched GO Terms from the molecular function ontology. Associated Treemap plots of the hierarchical clusters for GO Terms as grouped by biologic process, cellular component and molecular function are in Figure S2. More detail, including GO Term annotations, descriptions, enrichment, number of proteins (Uniprot Genes) involved from our dataset involved in each GO Term, and FDR‐qValues are in Table S3.

Slough tissue was first characterised by untargeted proteomics to determine the overall protein composition (Table S2). 11,058 peptide fragments (7302 unique peptide groups) corresponding to 1447 unique protein signatures were detected. To identify the biologic processes, molecular functions and cellular components that associated with protein features, abundant proteins identified across all samples were analysed using the Gene Ontology enRIchment analysis (GORILA) tool.^31^ This analysis confirmed that wound slough is significantly enriched for proteins involved in skin barrier formation, wound healing, regulation of blood clotting, as well as various immune functions including responding to bacteria, acute inflammatory responses, immune effector cell responses and humoral immunity (Figures 1A, S2A and Table S3). Overall, wound slough is mainly comprised of proteins derived from both intracellular and extracellular components, notably enriched for proteins specific to skin tissue, such as the cornified envelope and keratin filaments (Figures 1B, S2B and Table S3). Molecular pathway analysis also revealed slough to be enriched for proteins involved in ion and metabolite binding (Figures 1C and S2C). Collectively, identifying various collagens, extracellular matrix proteins, matrix metalloproteases, clotting factors and immune proteins within slough are consistent with proteins found in chronic wound tissue biopsies and exudative fluid.^9^, ^10^, ^11^, ^12^, ^13^, ^14^

**FIGURE 2:**
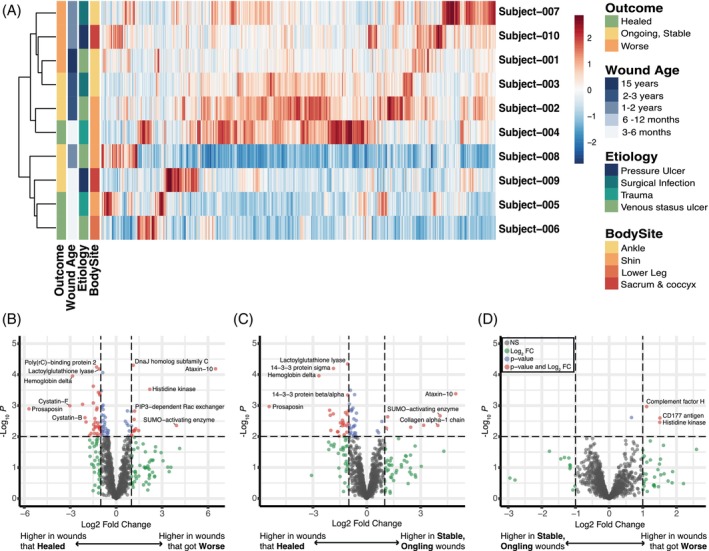
Wounds that go on to heal, are ongoing, or deteriorate are enriched for different proteins. (A) Heat map demonstrating each proteins' relative expression across all subjects demonstrates that samples largely grouped by the wounds relative age and outcome 3 months after the sample collection (e.g., whether the wound went on to heal, is ongoing but stable, or continued to deteriorate). (B–D) Subjects were grouped by the wound's outcome and groups were assessed for differential protein expression via DEqMS. Volcano plots indicating the proteins with significantly greater expression in; (B) wounds that continued to deteriorate vs. wounds that went on to heal; (C) wounds that were stable but on going vs. those that healed: (D) and wounds that continued to deteriorate vs. wounds that were ongoing but stable. Biologic functions of these highly expressed proteins were determined by the Gene Ontology Database. In brief, wounds that healed are enriched for proteins involved in skin barrier development, wound healing, blood clot formation, responses to bacteria and external stress. Wounds that deteriorated have higher expression of proteins involved in chronic inflammatory responses, the compliment cascade and a pseudomonas histone kinase. Figure S3 displays the volcano plot for differential protein expression in younger vs. older wounds.

To determine if the protein composition of slough is associated with clinical features and wound healing outcomes, hierarchical clustering using Euclidean distances was performed to identify patterns across the dataset. Clustering appeared to be driven by the wound age at sample collection and clinical outcome at 12 weeks‐post collection defined as healed, ongoing but stable, or deteriorating (Figure 2A). Proteins differentially abundant in healing wounds compared to those that were stable or deteriorated, were then determined using DEqMS.^33^ Forty‐eight proteins were differently abundant between healing wounds and deteriorating wounds, while 32 proteins were differently abundant between healing wounds and those that were ongoing yet stable (Figure 2B–D and Table S4). GO Enrichment Analysis^35^ shows that healing wounds are enriched for proteins involved in skin barrier development (e.g., cornifin‐B and 14‐3‐3 protein sigma), wound healing (e.g., beta‐2‐glycoprotein 1), blood clot formation (e.g., coagulation factor XIII) and responses to bacteria and external stress (e.g., immunoglobins, cystatin‐F and peroxiredoxin‐6). Conversely, deteriorating wounds are enriched for proteins involved in immune responses categorised as chronic inflammatory responses (e.g., AP‐1 complex subunit gamma‐1 and NLR family proteins) and the compliment cascade (e.g., Complement factor H). Finally, differential protein analysis between newer or older wounds found newer wounds (defined as being present for <1 year) are enriched in proteins involved in epithelial barrier formation and integrity (e.g., epithelial cell division and epithelial cell‐to‐cell adhesion), neutrophil degranulation and response to bacteria (Figure S3).

### 4.3 Wound slough is polymicrobial and associated with wound aetiology and body site

**FIGURE 3:**
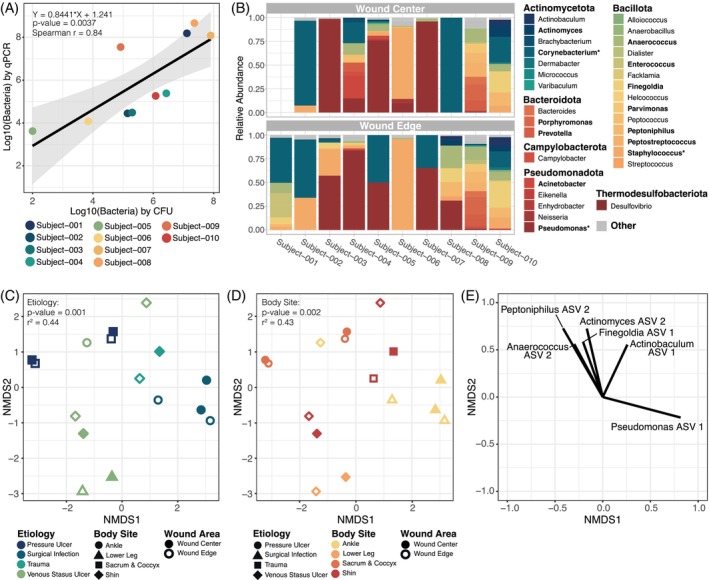
Microbial communities at a wound surface are largely dictated by the body site where the wound is located and the wound's aetiology. Swabs of the surface microbiome were collected from the wound edge and wound centre. Subject‐001 did not have a sample collected from the wound centre due to pain. (A) The number of bacteria per inch^2^ wound determined by quantitative‐PCR (qPCR) strongly corelates with the number of bacteria per inch^2^ wound detected through quantitative bacterial culture (measured in bacterial colony forming units [CFU]). Points are coloured by subject. (B) Relative abundance of bacterial genera on the surface of each subject's wound centre (top) and wound edge (bottom) based on high‐throughput sequencing of the bacterial 16S ribosomal gene. For each sample, bacterial taxa that were <1% abundant were grouped into the “Other” category along with any un‐classified bacterial sequences. Genera are grouped by the phyla in which they belong; Actinomycetota (blues), Bacillota (greens to orange‐yellows), Bacteroidota (oranges), Campylobacter (deep orange‐red), Pseudomonadota (reds) and Thermodesulfobacteriota (deep red). Relative abundance of bolded genera indicates that the genera comprises >10% of at least one sample. An * indicates that the genera comprises >30% of at least one sample. (C, D) The similarity of the microbial communities within each sample was evaluated with Bray–Curtis Dissimilarity Index. PERMANOVA assessment of the dissimilarity matrix with wound features indicated that wound surface microbial communities cluster by both the wound's aetiology (C) and the wounds location on the body (D; further detail in Table S6). Plots C and D are the same Non‐metric MultiDimensional Scaling (NMDS) ordination of the Bray–Curtis dissimilarity matrix but coloured differently to highlight the sample groupings by aetiology and body site, respectively. Microbiome samples did not cluster by whether the swab was taken from the wound edge or centre, the wounds age, or the wounds outcome 3 months after the sample collection (e.g., whether the wound went on to heal or did not). (E) A vector plot indicating the primary bacterial ASVs that dictated a points position in the NMDS plot C–D. Correlations between ASV abundance and a samples position were assessed with the Spearman correlation test. Only ASVs with *p* values <0.05 and *R* ^2^ > 0.2 are shown.

To assess the microbial bioburden of slough samples, swabs were collected from the wound surface, prior to washing and removal of slough via debridement. Bacterial bioburden was assessed by both quantitative bacterial culture and quantitative‐PCR of the bacterial 16S ribosomal RNA gene (Table S5). Slough bioburden was generally high across all samples ranging from 1.0 × 10^2^ to 8.0 × 10^7^ colony forming units (CFU) per inch^2^ wound and 4.2 × 10^3^ to 4.6 × 10^8^ bacteria per inch^2^ by qPCR. The quantity of bacteria determined by qPCR and the quantity of bacteria detected through quantitative bacterial culture are highly concordant (Figure 3A, spearman *r* = 0.84, *p* value <0.01).

To determine the composition and spatial variation of bacterial communities within slough, samples collected from slough at the edge and centre of the wound were assessed through high‐throughput sequencing of the bacterial 16S ribosomal RNA marker gene (Figures 3B and S4). Due to pain, subject‐001 did not have a sample collected from the wound centre for this analysis. The major bacterial genera detected were consistent with previous wound microbiome studies. Collectively, the most abundant taxa from wound samples include *Corynebacterium* spp., *Pseudomonas* spp. and *Staphylococcus* spp. (Figures 3B and S4). Overall microbiome community structure was generally consistent between the wound edge and wound centre (PERMANOVA *p* value >0.9; Table S6). However, in some cases microbiome composition drastically differed, such as in subject‐008, where a single species appears to dominate the wound centre while the wound edge harbours a much more diverse microbiome. Microbial communities dominated by few taxa within the centre of the wound more often occurred in the subjects with large (surface area >25 cm^2^), deep (>10 cm^3^) wounds.

The similarity of sample microbial community compositions was evaluated with the Bray–Curtis dissimilarity index (Figure 3C–E). Factors significantly associated with microbial community composition included the wound's aetiology and its location on the body (PERMANOVA *r* ^2^ = 0.44 and =0.43, respectively, both *p* values <0.01; Figure 3C, D and Table S6). Notably, community composition was not associated with spatial sampling at the wound edge or centre, or the outcome of the wound 3 months following sample collection (*p* values >0.05). The primary bacterial taxa whose abundance corelated with sample position in the NDMS plot belong to *Anaerococcus, Peptoniphilus, Actinomyces, Finegoldia, Actinobaculum* and *Pseudomonas* species (all spearman *r* ^2^ > 0.2, and *p* values <0.05; Figure 3E).

### 4.4 Detection of microbial aggregates in slough is highly variable

**FIGURE 4:**
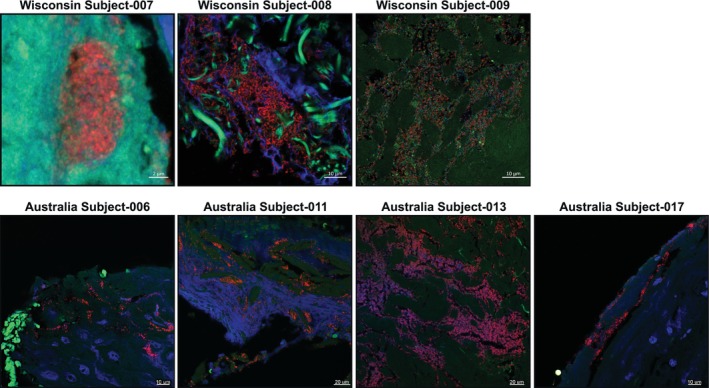
Confocal scanning laser microscopy of bacteria aggregates in slough. Formalin‐fixed, paraffin‐embedded (FFPE) slough samples were stained with a universal bacterial 16S rRNA probe (red) and for double stranded DNA (DAPI, blue) then visualised with confocal scanning laser microscopy (CSLM). Autofluorescence of the surrounding tissue was visualised in green. Only specimens with detected bacterial aggregates are shown here. Images from specimens with no bacterial aggregates are in Figure S5.

To evaluate potential commonalities in the microscopic structure of slough and associated microbial aggregates, slough samples were visualised using both confocal scanning laser microscopy (CLSM) and scanning electron microscopy (SEM). Overall, both techniques revealed slough to be highly variable in structure and composition. CLSM of slough histological sections revealed heterogenous, auto‐fluorescent fibrinous tissue and DNA (Figures 4 and S5). SEM showed complex milli‐, micron‐ and nano‐meter scale features on the slough surface, consistent with fibrin and collagen fibrils, fibres and bundles (Figure S6). One semifluid sample (Subject‐008) contained undefined crystalline structures.

Notably, microbes were only visible in three samples from the Wisconsin cohort, those from patients with the highest slough bioburden, and four of the Australian subjects (Figures 4 and S5). This indicates a lower sensitivity of microscopy‐based methods. Additionally, all three samples demonstrated different spatial distributions of microbes. Subject‐007 had small (~10 μm) aggregates embedded in tissue localised to a DNA‐rich, layered region of solid slough (CLSM; Figures 4 and S5). Subject‐008 had large (>50 μm) bacterial aggregates surrounded by extracellular DNA and putative collage fibres within the core of the semifluid slough, suggesting a biofilm community structure (CLSM; Figure 4). Subject‐009 had putative collagen bundles colonised with individual rods, cocci and lancet‐shaped bacteria (SEM; Figure S6). CLSM cross‐sections showed sparse bacteria in between tissue bundles (Figure 4).

### 4.5 Integrated analysis reveals key features of non‐healing wounds

**FIGURE 5:**
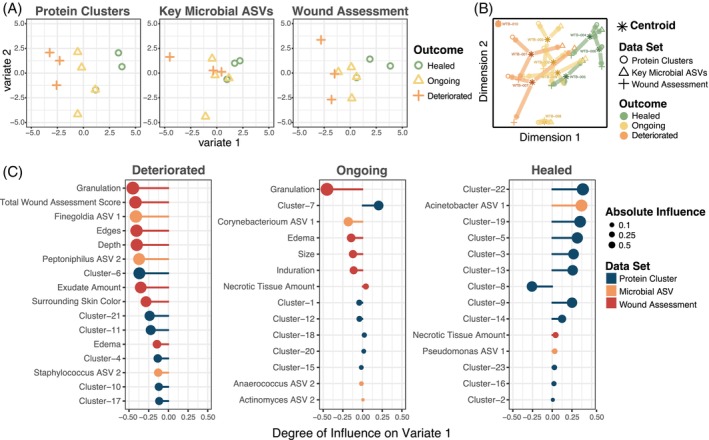
Chronic wounds that go on to heal can be distinguished from those that deteriorate via the proteomic, microbial and clinical features of slough. To predict the variables associated with wound healing, the protein cluster, microbial and the Bates‐Jensen Wound Assessment datasets were integrated into a supervised Partial Least Squares—Discriminant Analysis (PLS‐DA) via the MixOmics package. To simplify the proteomics dataset, proteins were grouped into 23 k‐means clusters via the gap‐stat method (Table S2). Since there was no significant difference in the microbial community composition at the wound edge or centre, samples were combined to create a summative wound slough microbiome for each subject. The “key” 14 microbial ASVs with >1% relative abundance in at least two subjects' slough samples were included in this integrative analysis (Table S7). (A) PLS‐DA plots for the protein cluster, microbial ASV and wound assessment data set, respectively. Each dataset contains variables that can distinguish chronic wounds that go on to heal from those that deteriorate. Outcome groups most clearly separate along variate 1 for each of the datasets. (B) PLS‐DA plot for all the data sets combined. The asterisk indicates the centroid position where the subject's slough sample falls considering variables from all three datasets. Arrows from the centroid indicate the direction that variables from each individual dataset pull the subject's datapoint. (C) Variable plots of the protein clusters (blue), microbial taxa (orange) and wound assessment criteria (red) that distinguish each outcome group along variate 1 of the PLS‐DA plots. A longer vector to the right indicates a variable with greater influence pulling samples to the right along the variate 1 axis. The enriched GO biologic processes for representative slough protein clusters that distinguish slough from wounds with each outcome are in Figure 6.

**FIGURE 6:**
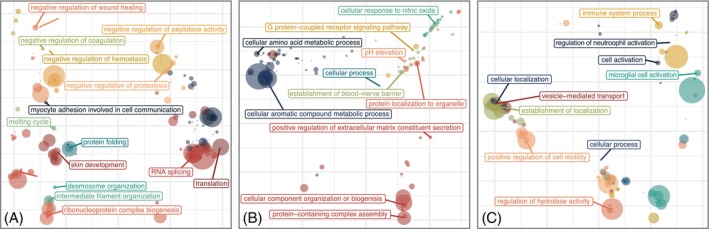
Slough from wounds that go on to deteriorate are enriched for immune activation and inflammatory immune responses. PLS‐DA analysis identified several protein clusters that can distinguish slough from wounds that went on to heal, were on going, or deteriorated (Figure 5). To determine the key biologic processes associated with each protein k‐means cluster, proteins within each cluster were submitted as unranked lists to the GO Enrichment analysis tool for evaluation with the PANTHER Overrepresentation test. Details for this analysis are included in Table S8. Plots display PCoA ordinations of significantly enriched biologic process GO Terms with in the top three protein clusters that distinguished wounds in each outcome. (A) Enriched GO biologic processes in wounds that went on to heal from heal (clusters 7, 1 and 12); (B) those that were ongoing yet stable (clusters 7, 1 and 12) and (C) wounds that deteriorated (clusters 6, 21 and 11). For each ordination the size of each point indicates the degree of significance for that terms enrichment in the dataset (the ‐log_10_[FDR *q*‐value]) and the distances between points represent the similarity between the biologic process GO Terms. Corresponding hierarchical clusters of these GO Terms enriched in wounds that healed, were ongoing, or deteriorated are in Figure S7. Figure S8 displays the significantly enriched GO biologic processes for all 23 k‐means clusters.

To predict the variables associated with wound healing outcome, an integrative analysis was pursued encompassing protein clusters, microbial taxa relative abundance and the numerical Bates‐Jensen Wound Assessment score. Datasets were integrated into a supervised Partial Least Squares—Discriminant Analysis (PLS‐DA).^37^ To reduce the complexity of the proteomics dataset, K‐means clustering was first performed. Further, the top 15 microbial ASVs with >1% relative abundance in at least two subject samples were included (Table S7). PLS‐DA revealed that the proteomic and microbial composition of slough and Bates‐Jensen scores can distinguish chronic wounds that go on to heal versus those that deteriorate (Figure 5A,B). Figure 4C illustrates the key variables that help distinguish each outcome group along variate 1 of the PLS‐DA plots. Wounds that deteriorated were associated with a higher total Bates‐Jensen Assessment score and sub‐scores (e.g., higher granulation tissue score, indicating smaller area of the wound bed covered by granulation tissue and poor vascular supply; higher wound edge score, indicating more well‐defined to thickened wound edge; as well as greater wound depth); increased abundance of anaerobic taxa (e.g., *Finegoldia* ASV1, *Peptoniphilus* ASV 2), and higher expression of protein clusters 6, 21 and 11. GO Enrichment Analysis revealed that these clusters were enriched for proteins involved in immune responses, particularly immune activation and responses to stimuli, cell motility and intracellular processes (Figure 6A). Conversely, wounds that went on to heal were associated with higher abundance of *Acinetobacter* ASV 1 and protein clusters 22, 19 and 5 (Figure 5C). These clusters were enriched for proteins involved in metabolic and biosynthetic processing, gene expression and regulation (including negative regulation) of wound healing and responses to stress (Figures 6C, S7 and S6 and Table S8). Overall, the findings of this integrative analysis highlight potentially fundamental differences in the microbial and proteomic composition of slough from wounds that go on to heal compared to those at higher risk for progression.

## 5 DISCUSSION

Slough is a highly common and burdensome feature of wounds. However, its definition and composition remain poorly characterised. This pilot study aimed to characterise the host and microbial elements of slough across a variety of wound aetiologies. We also sought to identify key factors within slough associated with wound healing trajectories. Our findings demonstrate that, (i) the microscopic structure of slough is heterogenous and unique to each wound; (ii) across subjects wound slough is composed of proteins involved in the structure and formation of the skin, blood clot formation and various immune responses; (iii) the microbial community composition is diverse and corresponds to the wound's aetiology and location on the body and (iv) the composition of slough is associated with wound healing outcomes. Collectively, these findings underscore how the composition of slough itself may be useful for developing microbial and proteomic biomarkers prognostic of wound healing trajectories.

The clinical presentation of slough is highly variable. Slough can range in colour from pale yellow to yellow‐green, tan, brown, or black to resemble eschar. It can also range in texture from mucous‐wet to thick and fibrinous, and range from loosely to firmly attached^6^, ^8^ As expected with this variable clinical presentation, confocal and scanning electron microscopic imaging reveals slough to be microscopically heterogenous and different across wounds (Figures S5 and S6). However, as noted by others, slough's intrinsic gelatinousness consistency makes it easy to perturb and difficult to fix for microscopic assessment.^39^ This likely limits our ability to ascertain additional three‐dimensional features within slough that may be pertinent to the wound surface environment.

Microbial biofilm is thought to be highly integrated within wound slough.^22^ In the clinical setting wound slough is often mistaken for microbial biofilm.^16^, ^22^ To address this, several studies have proposed wider adoption of culture based, molecular (i.e., quantitative‐PCR) and microscopic techniques into diagnostic practice.^40^, ^41^, ^42^, ^43^ At the time of sample collection, only one of the 10 subjects was diagnosed with a current wound infection and five had a history of infection in the sampled wound (Table 1). However, SEM and CLSM imaging detected microbes in only three of nine samples tested (Figures S5 and S6). Interestingly, subject‐009, who had no record of current or previous wound infection, was the only subject to have microbes visualised via both SEM and CLSM. This speaks to the difficulty in identifying biofilm or even the presence of single cells of bacteria using microscopy techniques as more sensitive molecular methods indicated every sample contained a considerable bioburden of bacteria. Further, the detection rates for microbial biofilm in this study are notably lower than previously reported for chronic wound samples.^42^, ^43^, ^44^ This could be due to a number of factors, including spatial heterogeneity of bacterial aggregates across the wound surface. Indeed, to saturate sampling efforts hundreds of slides and images would need to be obtained. To improve detection rates the incorporation of methods that increase specificity of bacterial detection such as immunogold labelling or gold in situ hybridization could be applied, but remain impractical for routine clinical evaluation.^45^, ^46^

The quantity of bacteria determined via qPCR correlates with the bacterial burden as determined by quantitative culture (Figure 3A).The reference standard for clinical definition of a wound infection is 10^5^ or more cultured colony forming units (CFU)/ml.^40^ By that metric, eight of the 10 subjects meet definition for clinical infection, despite an absence of clinical sign of infection (Figure 3A). Indeed, only one subject had a diagnosed infection. While the use of qPCR for detecting bacterial bioburden is more sensitive, particularly for patients like subject‐009 whose wounds may contain more anaerobic or difficult to culture bacteria (Figure 3A, B), this data suggests the use of such cutoffs are complicated and should be used with caution. Indeed, wounds with high bacterial bioburden can go on to heal without intervention with antibiotics.

Isolation and identification of bacteria from all subjects through both microbial culture and 16S sequencing underscores that even in the absence of a clinical wound infection, slough contains complex microbial communities (Figure 3A, B and Table S5). Previous study evaluating the influence of sharp debridement on the wound microbiome further has shown that these microbes are likely highly integrated within and throughout wound slough.^17^ Here, the most frequently isolated via microbial culture were *Corynebacterium, Staphylococcus* and *Pseudomonas* species (Table S6). *Corynebacterium, Staphylococcus* and *Pseudomonas* were also the most abundant taxa identified via 16S profiling, comprising at least 30% of the microbial community in slough three of the 10 subjects, respectively (Figure 3B). Across wound aetiologies, from multiple studies, *Corynebacterium, Staphylococcus* and *Pseudomonas* species appear to be the most abundant taxa within the chronic wound microbiome (Figure 3B).^17^, ^18^, ^19^, ^47^ Contradicting some previous reports,^17^, ^19^, ^47^ we find slough microbial community composition to be associated wound aetiology and location on the body (Figure 3C–E and Table S6). The microbiome of healthy intact skin naturally varies across body sites due to differences in the physiologic characteristics of the local skin environment.^48^, ^49^, ^50^, ^51^, ^52^ It is plausible that these variations in the microbiome of the surrounding skin influence the community structures within wound slough.

In terms of the human components of the wound, there are only a handful of reports on the proteomic composition of tissue biopsies, granulation tissue and exudative fluid from chronic pressure ulcers and diabetic wounds.^9^, ^10^, ^11^, ^12^, ^13^, ^14^ Broadly speaking, these studies find fluid and tissue from these wounds to contain various types of collagen, extracellular matrix proteins, matrix metalloproteases, clotting factors and proteins generally related to innate and acute immune responses.^9^, ^10^, ^11^, ^12^, ^13^, ^14^ Although this is a pilot study, to our knowledge, this is the first work to specifically evaluate the proteomic composition of wound slough. In line with these previous studies, slough from chronic wounds is primarily composed of keratin and various types of collagen, extracellular matrix proteins, matrix metalloproteases, clotting factors and immune response proteins (Table S2 and Figure 1). More specifically, slough is enriched for proteins involved in skin barrier integrity and formation, wound healing and immune functions ranging from innate compliment activation to acute responses to stimuli (e.g., to bacteria) and humoral immune responses (Figures 1 and S2). The high prevalence of intracellular and skin associated proteins combined with the relative absence of enrichment for vascular and angiogenic pathways supports that hypothesis that slough is largely devitalized tissue. However, many of these proteins may be functional in this environment. Further, the collective abundance of proteins associated with inflammatory cells such as neutrophils, underscores the leading theory that slough is a biproduct of prolonged inflammatory process.^6^, ^7^

Of the three main data sets assessed (proteomics, microbiome, and the Bates Jensen Wound Assessment), the proteomics dataset had the strongest associations with wound healing outcome. When assessed independently, Bates‐Jensen Wound Assessment Tool (BWAT) scores were not significant for wounds that deteriorated, nor were there associations between microbiome composition and wound outcome (Table 2 and Figure 3). Although these analyses were likely limited due to low subject numbers, differential Protein Expression Analysis (DeqMS)^33^ found wounds that went on to heal were enriched for proteins involved in skin barrier development, wound healing, blood clot formation and responses to bacteria and external stress (Figure 2B–D and Table S4). Conversely, deteriorating wounds were enriched for proteins involved in immune responses categorised as chronic inflammatory responses and the compliment cascade. Of the proteins enriched in wounds that deteriorated; AP‐1 is a notable biphasic regulator of wound healing^53^; NLR family proteins and Caveolase‐associated protein 1 have been associated with impaired wound healing in murine models^54^, ^55^, ^56^ and CD177, compliment factor H and vasodilator‐stimulated phosphoprotein have also been noted to be elevated in chronic wound fluid and/or tissue.^11^, ^12^ To identify variables associated with wound healing we incorporated proteomics, microbiome and the BWAT score datasets into a supervised Partial Least Squares—Discriminant Analysis (PLS‐DA).^37^ In this model, slough from wounds that healed were enriched for proteins involved in regulation, particularly negative regulation, of immune responses and wound healing as well as the aerobic microbial taxa *Acinetobacter* (Figures 5 and 6). Conversely, wounds that deteriorated contained slough enriched with inflammatory proteins, particularly those involved in immune activation, responding to stimuli and chronic inflammation. Wounds that deteriorated were also associated with greater abundance of anaerobic microbial taxa, *Finegoldia* and *Peptoniphilus*, as well as higher Bates‐Jensen wound assessment scores, indicating a more severe wound state.

Overall, our exploratory model's findings are consistent with related literature. For instance, they underscore BWAT's clinical utility in a well‐rounded wound evaluation, and suggest that high sub‐scores for granulation, wound edge, wound depth and exudate amount may hold the strongest predictive potential for identifying a wound likely to deteriorate (Figure 5).^25^, ^38^, ^57^ From a microbiological perspective, high abundance of anaerobic taxa and select *Staphylococci* species are frequently associated with impaired wound healing and poor outcomes.^17^, ^18^, ^27^, ^58^ Similar proteomic investigations with wound fluid and tissue biopsies also find elevated inflammatory proteins and enrichment of proteases and matrix metalloproteinases in wounds that do poorly as well as enrichment of extracellular matrix proteins and keratin in healing wounds.^10^, ^12^ This study demonstrates that slough, which is often regularly debrided as a part of standard care, provides a readily available, underutilised, high protein concentration biomarker reservoir. Most of the proteins identified through independent DeqMS assessment also fall within the protein clusters that distinguish between healed and non‐healing wounds in the comprehensive model (Figures 2 and 5 and Tables S2, S4 and S8), suggesting these identified proteins have the greatest potential as biomarker targets to predict wound healing.

The primary limitation of this pilot study is the small number of subjects enrolled. Future investigations intend to expand upon these methods, potentially with even more targeted proteomic and microbiologic approaches, to validate the predicted features associated with wound healing outcome in a larger cohort. In future studies we aim to sample slough from various depths of the wound to obtain a more in‐depth analysis. We also aim to intentionally sample from multiple regions of a wound to obtain a topographical map of the proteins and microbes within slough across a wound's surface. Another limitation was that we were unable to collect tissue samples from the underlying wound bed itself, after removal of slough. Future studies should consider collecting both slough and wound tissue samples to understand the proportion of slough proteins that overlap with proteins also found in the wound bed. There were also several factors that inhibited the microscopic detection of microorganisms via SEM and CLSM. For instance, initial cleansing of the wound with soap and water prior to debridement per standard of care, may have removed superficial microbial aggregates. With SEM, there are no efficient algorithms for distinguishing individual microbes or microbial biofilm from background collagen fibres and tissue. The heterogeneous and often gelatinous texture of slough along with the ability to only view a histological cross‐section, may have also limited microbial detection via CLSM. Our microbial assessment with 16S amplicon sequencing only provides genus level resolution, and not all species within a genus have propensity to cause infection (e.g., *Staphylococcus aureus* vs. *Staphylococcus hominis*). Evaluating chronic wound metagenomes would provide species level resolution and detect the presence of virulence and antibiotic resistance genes. However, to date there are very few investigations into wound microbial metagenomes as this method remains limited due to cost.^18^

In conclusion, slough is an underutilised reservoir for potential microbial and proteomic biomarkers. To our knowledge this is the first study to integrate clinical wound assessment, microbiome and proteomic data into a single assessment for the prediction of wound healing outcome. Future studies intend to utilise these and similar methods to further explore the biomarkers within slough in a larger cohort with appropriate statistical power. Utilisation of a comprehensive patient‐centred assessment will lead to more effective identification of high‐risk patients wounds for triage into specialty care, ultimately, reducing the healthcare, financial and personal burden of living with hard to heal wound.

## AUTHOR CONTRIBUTIONS

Conceptualization: Lindsay R. Kalan, Karen Ousey, Terry Swanson, Gregory Schultz. Methodology: Lindsay R. Kalan, Thomas Bjarnsholt, Matthew Malone, Elizabeth C. Townsend, J. Z. Alex Cheong, Michael Radzietza, Blaine Fritz. Analysis: Lindsay R. Kalan, Elizabeth C. Townsend, J. Z. Alex Cheong, Thomas Bjarnsholt, Matthew Malone, Michael Radzietza, Blaine Fritz. Investigation: Lindsay R. Kalan, Angela L. F. Gibson, Matthew Malone, Thomas Bjarnsholt, Elizabeth C. Townsend, J. Z. Alex Cheong, Michael Radzietza, Blaine Fritz. Resources: Lindsay R. Kalan, Angela L. F. Gibson, Matthew Malone, Thomas Bjarnsholt. Writing—Original Draft: Elizabeth C. Townsend, J. Z. Alex Cheong, Lindsay R. Kalan, Angela L. F. Gibson. Writing—Review & Editing: Elizabeth C. Townsend, J. Z. Alex Cheong, Gregory Schultz, Matthew Malone, Thomas Bjarnsholt, Michael Radzietza, Blaine Fritz, Karen Ousey, Terry Swanson, Angela L. F. Gibson, Lindsay R. Kalan. Visualisation—Elizabeth C. Townsend, J. Z. Alex Cheong, Michael Radzietza, Blaine Fritz. Supervision—Lindsay R. Kalan, Angela L. F. Gibson, Thomas Bjarnsholt, Matthew Malone.

## FUNDING INFORMATION

This study has been supported by an unrestricted educational grant from Coloplast, Convatec, Hartmann, L&R and Medline. This study was also supported by grants from the National Institutes of Health (NIGMS R35 GM137828 [Lindsay R. Kalan]). The content is solely the responsibility of the authors and does not necessarily represent the official views of the National Institutes of Health.

## Supporting information


**FIGURE S1.** Photos of subject wounds before debridement procedure.

**FIGURE S2.** Treemap plots displaying hierarchical clusters of the significantly enriched gene ontology (GO) terms grouped by biologic processes (A) molecular functions (B) and cellular components (C) within wound slough. The most abundant proteins across all slough debridement tissue samples were input as a ranked list to the Gene Ontology enRIchment analysis (GORILA) and visualisation tool.^31^ Treemap plots were then created from these results with rrvgo.^32^ Figure 1 displays corresponding PCoA plots of the enriched GO Terms. More detail, including GO Term annotations, descriptions, enrichment, number of proteins (Uniprot Genes) involved from our dataset involved in each GO Term, and FDR‐qValues are in Table S3.

**FIGURE S3.** Chronic wounds present <1 year are enriched for proteins involved in epithelial barrier formation, neutrophil degranulation and response to bacteria. Conversely, wounds present for more than 1 year are enriched for proteins involved in iron sequestration and tRNA metabolism. Subjects were grouped the age of the wound at the time of sample collection. Wounds present for <1 year were considered “young”, and those present for more than 1 year were considered “old.” Groups were assessed for differential protein expression via DEqMS. This volcano plot displays the proteins with significantly greater expression in younger or older wounds.

**FIGURE S4.** The Abundance of key bacterial taxa is similar across wound slough from two distinct subject cohorts from Wisconsin and Australia. Datasets were generated using amplicon sequencing of the V4 (panel A, Wisconsin) or V1V3 (panel B, Australia) regions of the 16S rRNA gene, and were thus analysed separately. ASVs were summed at the genus level. Note that the taxonomic resolution for classification may differ by amplicon region. Genera are shown if present at above 5% relative abundance in at least one specimen and are ordered by mean relative abundance across all specimens within a dataset. Subjects are ordered by average linkage hierarchical clustering of Bray‐Curtis dissimilarities. In the Wisconsin cohort (panel A), subject taxa profiles are averaged from multiple specimens.

**FIGURE S5.** Confocal scanning laser microscopy of slough samples without bacterial aggregates. Formalin‐fixed, paraffin‐embedded (FFPE) slough samples were stained with a universal bacterial 16S rRNA probe (red) and for double stranded DNA (DAPI, blue) then visualised with confocal scanning laser microscopy (CSLM). Autofluorescence of the surrounding tissue was visualised in green. The specimens with detected bacterial aggregates are shown in Figure 4. Here are the remaining specimens from both patient cohorts that did not have identifiable bacterial aggregates.

**FIGURE S6.** Scanning electron microscopy finds slough to be variable in structure and unique to the subject. Debrided slough samples were evaluated via scanning electron microscopy (SEM). Subjects‐004, ‐005 and ‐006 did not have enough debridement tissue for SEM. One subject, subject‐009 had visible microorganisms on SEM. A majority of specimens were fibrous in appearance, while one specimen had crystalline structures.

**FIGURE S7.** Treemap plots displaying hierarchical clusters of significantly enriched biologic process gene ontology (GO) terms in wounds that went on to heal (A), were ongoing yet stable (B) or deterioriated (C) 3 months following sample collection. PLS‐DA analysis identified several protein clusters that can distinguish slough from wounds that went on to heal, were on going, or deteriorated (Figure 5). This is a companion plot to Figure 6. Treemaps display; (A) Enriched GO biologic processes in wounds that went on to heal from heal (clusters 7, 1 and 12); (B) those that were ongoing yet stable (clusters 7, 1 and 12) and (C) wounds that deteriorated (clusters 6, 21 and 11). Representation of the key GO terms in all 23 clusters is in Figure S8.

**FIGURE S8.** Enriched GO biologic processes for each of the 23 k‐means protein clusters. To determine the key biologic processes associated with each protein k‐means cluster, proteins within each cluster were submitted as unranked lists to the GO Enrichment analysis tool for evaluation with the PANTHER Overrepresentation test. Details for this analysis are included in Table S8. This figure displays the top 25 most enriched GO biologic process each of the 23 k‐means protein clusters. For each biologic processes are ordered from most significantly enriched (smallest FDR q‐value) at the top to least enriched (largest FDR q‐value) at the bottom. Colour of the point indicates the broader biologic classification.

**TABLE S1.** Detailed subject and wound characteristics. Ten subjects with chronic or slow to heal wounds of various aetiologies were enrolled from the UW‐Health Wound Care Clinic. Wounds were evaluated with the Bates‐Jensen Wound Assessment Tool. Information on the wound and patient comorbidities were extracted from the medical record at the time of sample collection. Information on whether the wound went on to heal, was ongoing yet clinically stable, or deteriorated 3 months following sample collection was also recorded. This table serves as a compliment to Tables 1 and 2, providing subject level detail on the wound and patient comorbidities.
**TABLE S2.** Proteomic composition of wound slough. Normalised and means centred protein peptide abundance within each subject's wound slough. Description, species of origin (eg. Homosapiens or bacteria), broad GO biological process, GO cellular component, GO molecular function, WikiPathways, Reactome Pathways and KEGG pathways for each protein peptide accession are also included. To simplify the proteomics dataset for integrative PLS‐DA analysis proteins were grouped into 23 clusters via the gap‐stat method. The Kmeans cluster in which the protein falls is also indicated.
**TABLE S3.** Data‐frame of the GO Terms that are significantly enriched in chronic wound slough. Debrided slough tissue was sent for proteomic characterisation via mass spectrometry. (A) The most abundant proteins across all samples were input as a ranked list to the Gene Ontology enRIchment analysis (GORILA) and visualisation tool.^31^ Significantly enriched GO Terms are listed with their GO Term annotations, descriptions, enrichment, number of proteins (Uniprot Genes) involved from our dataset involved in each GO Term, and FDR‐qValues. Visual representations of this data can be found in Figures 1 and S2.
**TABLE S4.** Proteins with significantly greater expression subjects grouped by outcome or wound age. Subjects were grouped by the wound's outcome (healed, ongoing, deteriorated), or wound age (young [wounds present <1 year], or old [wounds present >1 year]) and groups were assessed for differential protein expression via DEqMS. Only proteins with significantly greater expression (log_2_ Fold change >1 and log_10_ Limma *p* value <10^−2^) are displayed. Benjamini–Hochberg method adjusted *p* values are displayed in the adj.P.val column. Details on the protein description and associated GO Terms, WikiPathways, Reactome Pathways and KEGG pathways are included. Volcano plots indicating the proteins with significantly greater expression in each of the associated group comparisons are in Figures 2B–D and S5.
**TABLE S5.** Bacterial bioburden and Identification of Cultured bacteria from the wound surface. Swabs of the wound slough microbiome were collected into either DNA/RNA Shield or liquid ames broth. Bacterial DNA was extracted from the samples collected into DNA/RNA Shield, and bacterial bioburden was assessed through quantitative PCR of the 16S ribosomal gene. Samples collected into liquid ames broth were plated on to blood agar and grown overnight at 37C for quantitative bacterial culture. Individual bacterial colonies with distinct morphologies were isolated and grown overnight. To identify the genus of these isolates, bacterial DNA was extracted and sent for sanger sequencing of the 16S bacterial ribosomal gene. Genera of successfully identified isolates are listed.
**TABLE S6.** Wound microbial community composition associates with the wound's aetiology and location on the body. Similarity of slough microbial compositions were evaluated with the Bray–Curtis dissimilarity metric. Associations between wound features and microbiome composition were assessed with univariate permutational multivariate analysis of variance (PERMANOVA). Each PERMANOVA was run considering the marginal effects of terms with 9999 permutations using the Adonis 2 *r* package. Benjamini‐Hochberg procedure was then used to correct *p* values for multiple hypothesis testing. * Indicates p‐value <0.05. ** Indicates *p* value <0.01.
**TABLE S7.** Relative abundance for key microbial ASV's in wound slough. Since there was no significant difference in the microbial community composition at the wound edge or centre (PERMANOVA *p* value >0.9, Table S6), samples were combined to create a summative wound slough microbiome for each subject. Relative abundance of each ASV in the wound centre and wound edge were averaged. This table displays the 14 microbial ASVs with >1% relative abundance in at least two summative subject slough microbiome samples, which were included in the integrative PLS‐SA analysis. Although not included in the PLS‐DA analysis, the relative abundance of all other taxa, which were either only present in samples from that subject, or present at <1% is also indicated to highlight the proportion of taxa within a subject that were of low abundance or unique to that subject.
**TABLE S8.** Most enriched GO biologic processes for each of the 23 k‐means protein clusters. To determine the key biologic processes associated with each protein k‐means cluster, proteins within each cluster were submitted as unranked lists to the GO Enrichment analysis tool for evaluation with the PANTHER Overrepresentation test. This table depicts the 25 most significantly enriched GO biologic processes for each protein cluster and includes the associated GO Terms, the broader classification, number of protein IDs in *Homo sapiens* reference database, number of IDs in uploaded K‐means cluster, the expected number of IDs, fold enrichment, Fisher's exact *p* value and Benjamini–Hochberg procedure false discovery rate (FDR) if it was able to be calculated. To determine the most enriched biologic processes, terms by the smallest to largest FDR, followed by smallest to largest *p* value if FDR was unable to be calculated. A rank of 1 indicates that it was the most enriched biologic process in the protein cluster. Table S2 includes details on the proteins within each cluster.

## Data Availability

Sequence reads for this project can be found under NCBI BioProject PRJNA1021648. Code for analysis and generation of figures can be found on GitHub at https://github.com/Kalan-Lab/Townsend_etal_WhatIsSlough.
