# Supplementary material for: What is slough? Defining the proteomic and microbial composition of slough and its implications for wound healing
Source: Wound Repair Regen. 2024 Apr 1;32(6):783–98. doi: 10.1111/wrr.13170 (PMC11442687; doi:10.1111/wrr.13170)
Supplement: Supplementary file 2 — FIGURE S2. Treemap plots displaying hierarchical clusters of the significantly enriched gene ontology (GO) terms grouped by biologic processes (A) molecular functions (B) and cellular components (C) within wound slough. The most abundant proteins across all slough debridement tissue samples were input as a ranked list to the Gene Ontology enRIchment analysis (GORILA) and visualisation tool. 31 Treemap plots were then created from these results with rrvgo. 32 Figure 1 displays corresponding PCoA plots of the enriched GO Terms. More detail, including GO Term annotations, descriptions, enrichment, number of proteins (Uniprot Genes) involved from our dataset involved in each GO Term, and FDR‐qValues are in Table S3. [file WRR-32-783-s003.pdf]

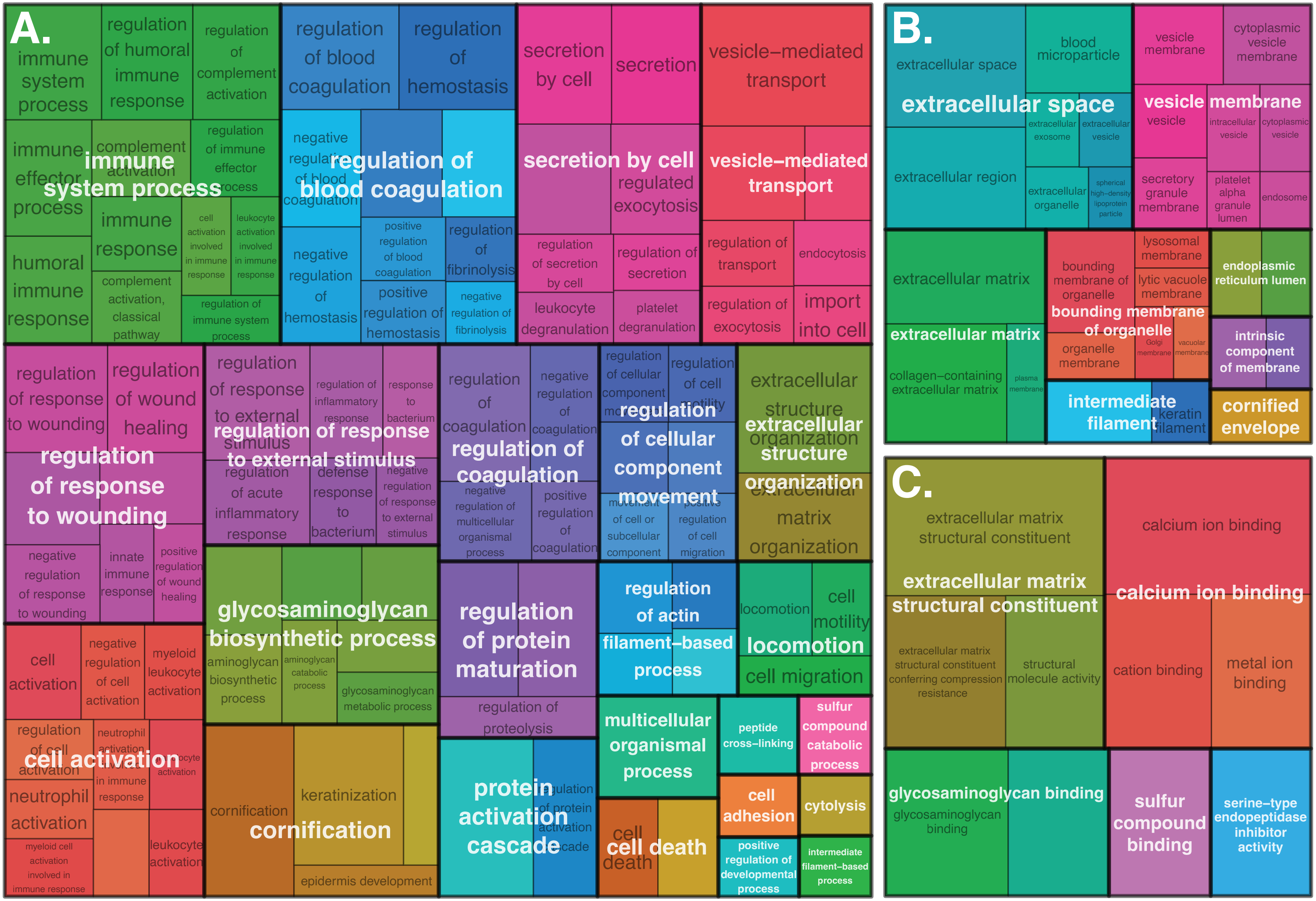

Supplemental Figure 2: Treemap plots displaying hierarchical clusters of the significantly enriched gene ontology (GO) terms grouped by biologic processes (A) molecular functions (B) and cellular components (C) within wound slough. The most abundant proteins across all slough debridement tissue samples were input as a ranked list to the Gene Ontology enRIchment analysis (GORILA) and visualization tool.29 treemap plots were then created from these results with rrvgo1 Figure 1 displays corresponding PCoA plots of the enriched GO Terms. More detail, including GO Term annotations, descriptions, enrichment, number of proteins (Uniprot Genes) involved from our dataset involved in each GO Term, and FDR-qValues are in Supplemental Table 3.
