# Supplementary material for: What is slough? Defining the proteomic and microbial composition of slough and its implications for wound healing
Source: Wound Repair Regen. 2024 Apr 1;32(6):783–98. doi: 10.1111/wrr.13170 (PMC11442687; doi:10.1111/wrr.13170)
Supplement: Supplementary file 6 — FIGURE S6. Scanning electron microscopy finds slough to be variable in structure and unique to the subject. Debrided slough samples were evaluated via scanning electron microscopy (SEM). Subjects‐004, ‐005 and ‐006 did not have enough debridement tissue for SEM. One subject, subject‐009 had visible microorganisms on SEM. A majority of specimens were fibrous in appearance, while one specimen had crystalline structures. [file WRR-32-783-s004.pdf]

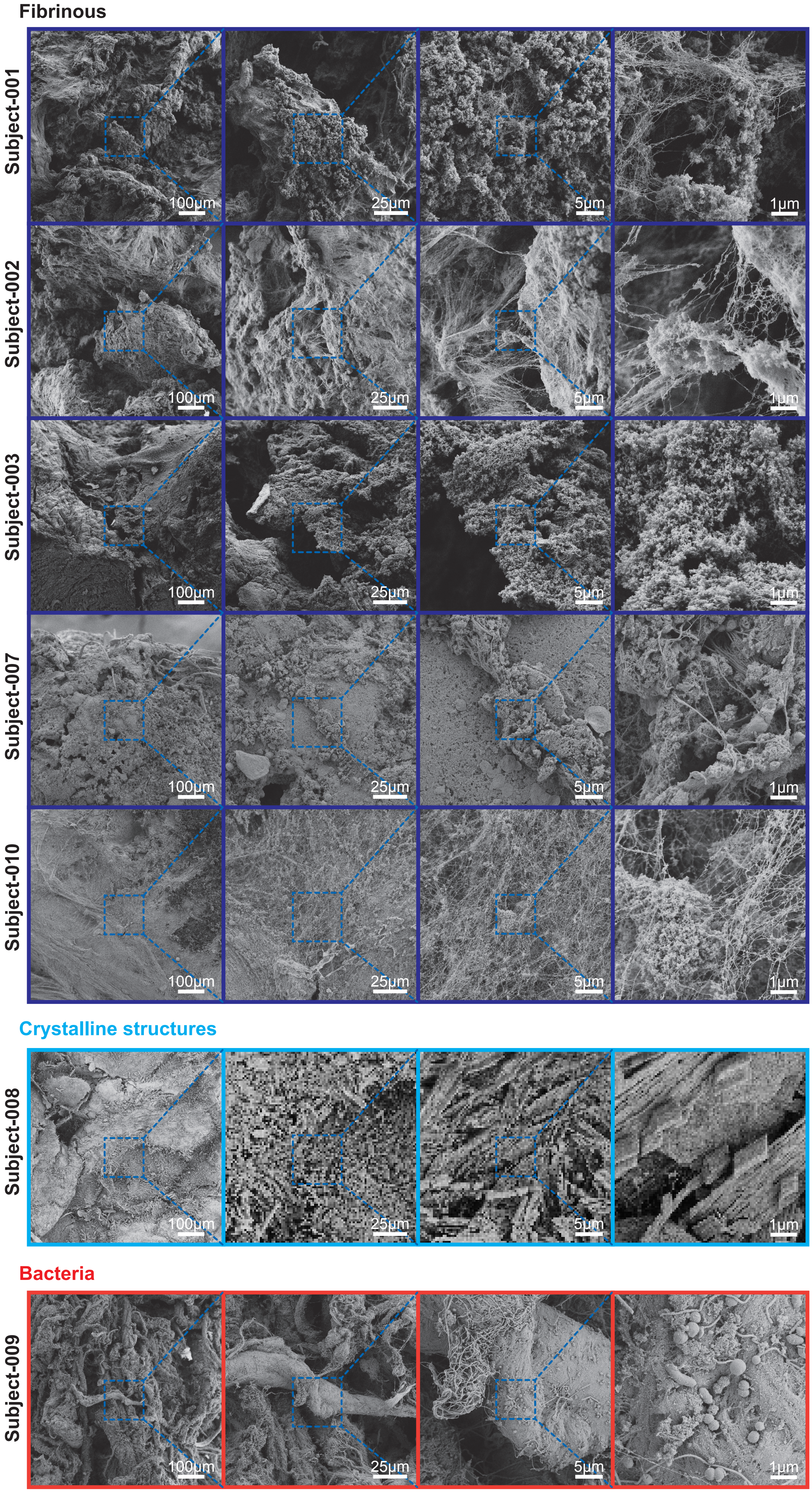

Supplemental Figure 6: Scanning electron microscopy finds slough to be variable in structure and unique to the subject. Debrided slough samples were evaluated via scanning electron microscopy (SEM). Subjects-004, -005, and -006 did not have enough debridement tissue for SEM. One subject, subject-009 had visible microorganisms on SEM. A majority of specimens were fibrous in appearance, while one specimen had crystalline structures.
