# Supplementary material for: What is slough? Defining the proteomic and microbial composition of slough and its implications for wound healing
Source: Wound Repair Regen. 2024 Apr 1;32(6):783–98. doi: 10.1111/wrr.13170 (PMC11442687; doi:10.1111/wrr.13170)
Supplement: Supplementary file 7 — FIGURE S7. Treemap plots displaying hierarchical clusters of significantly enriched biologic process gene ontology (GO) terms in wounds that went on to heal (A), were ongoing yet stable (B) or deterioriated (C) 3 months following sample collection. PLS‐DA analysis identified several protein clusters that can distinguish slough from wounds that went on to heal, were on going, or deteriorated (Figure 5). This is a companion plot to Figure 6. Treemaps display; (A) Enriched GO biologic processes in wounds that went on to heal from heal (clusters 7, 1 and 12); (B) those that were ongoing yet stable (clusters 7, 1 and 12) and (C) wounds that deteriorated (clusters 6, 21 and 11). Representation of the key GO terms in all 23 clusters is in Figure S8. [file WRR-32-783-s009.pdf]

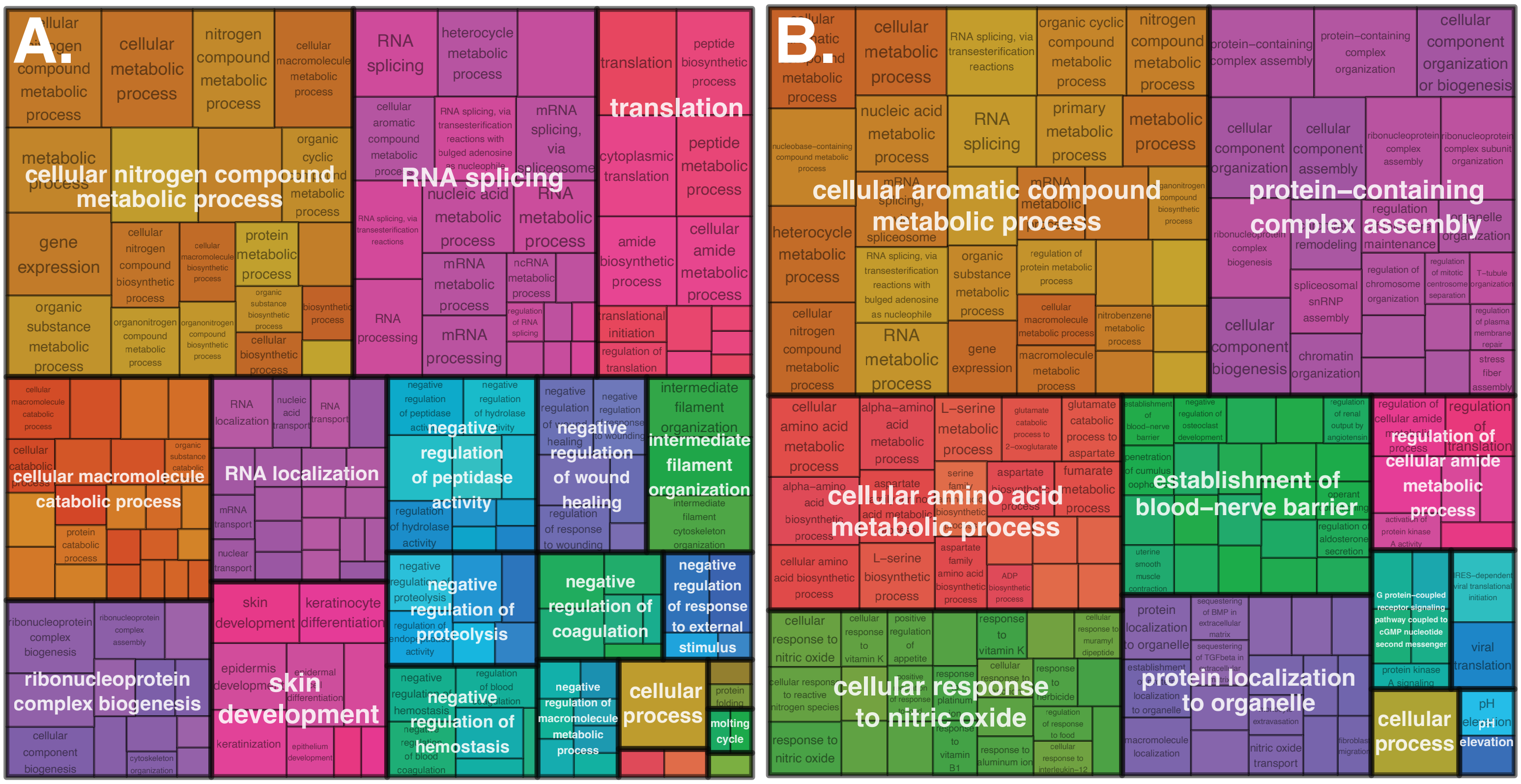

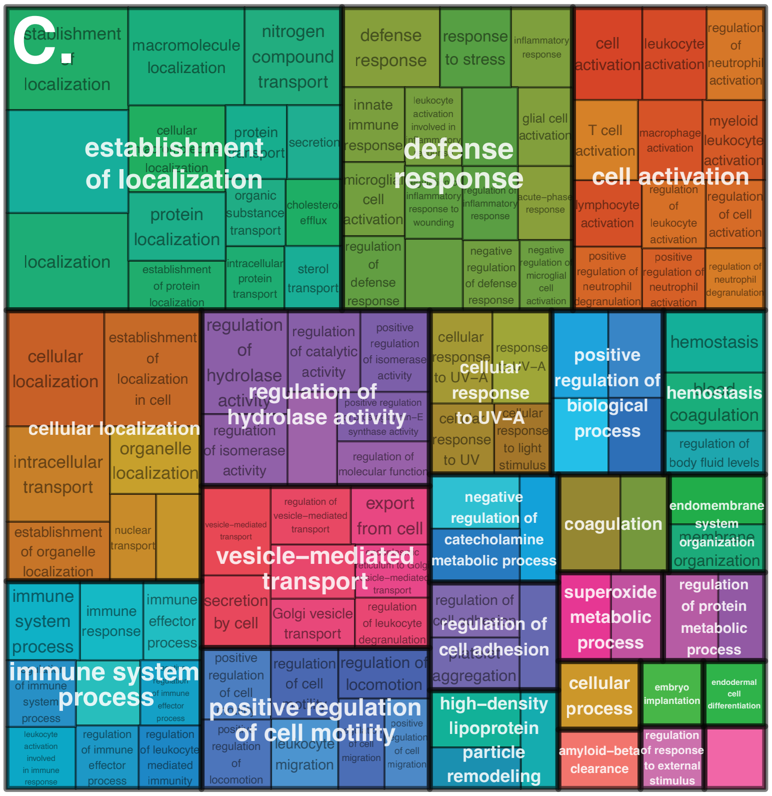

Supplemental Figure 7: Treemap plots displaying hierarchical clusters of significantly enriched biologic process gene ontology (GO) terms in wounds that went on to heal (A), were ongoing yet stable (B) or deterioriated (C) 3 months following sample collection. PLS-DA analysis identified several protein clusters that can distinguish slough from wounds that went on to heal, were on going, or deteriorated (Fig. 5). This is a companion plot to figure 6. Treemaps display; A) Enriched GO biologic processes in wounds that went on to heal from heal (clusters 7, 1, and 12); B) those that were ongoing yet stable (clusters 7, 1, and 12); and C) wounds that deteriorated (clusters 6, 21, and 11). Representation of the key GO terms in all 23 clusters is in supplemental figure 8.
