# Supplementary material for: What is slough? Defining the proteomic and microbial composition of slough and its implications for wound healing
Source: Wound Repair Regen. 2024 Apr 1;32(6):783–98. doi: 10.1111/wrr.13170 (PMC11442687; doi:10.1111/wrr.13170)
Supplement: Supplementary file 8 — FIGURE S8. Enriched GO biologic processes for each of the 23 k‐means protein clusters. To determine the key biologic processes associated with each protein k‐means cluster, proteins within each cluster were submitted as unranked lists to the GO Enrichment analysis tool for evaluation with the PANTHER Overrepresentation test. Details for this analysis are included in Table S8. This figure displays the top 25 most enriched GO biologic process each of the 23 k‐means protein clusters. For each biologic processes are ordered from most significantly enriched (smallest FDR q‐value) at the top to least enriched (largest FDR q‐value) at the bottom. Colour of the point indicates the broader biologic classification. [file WRR-32-783-s007.pdf]

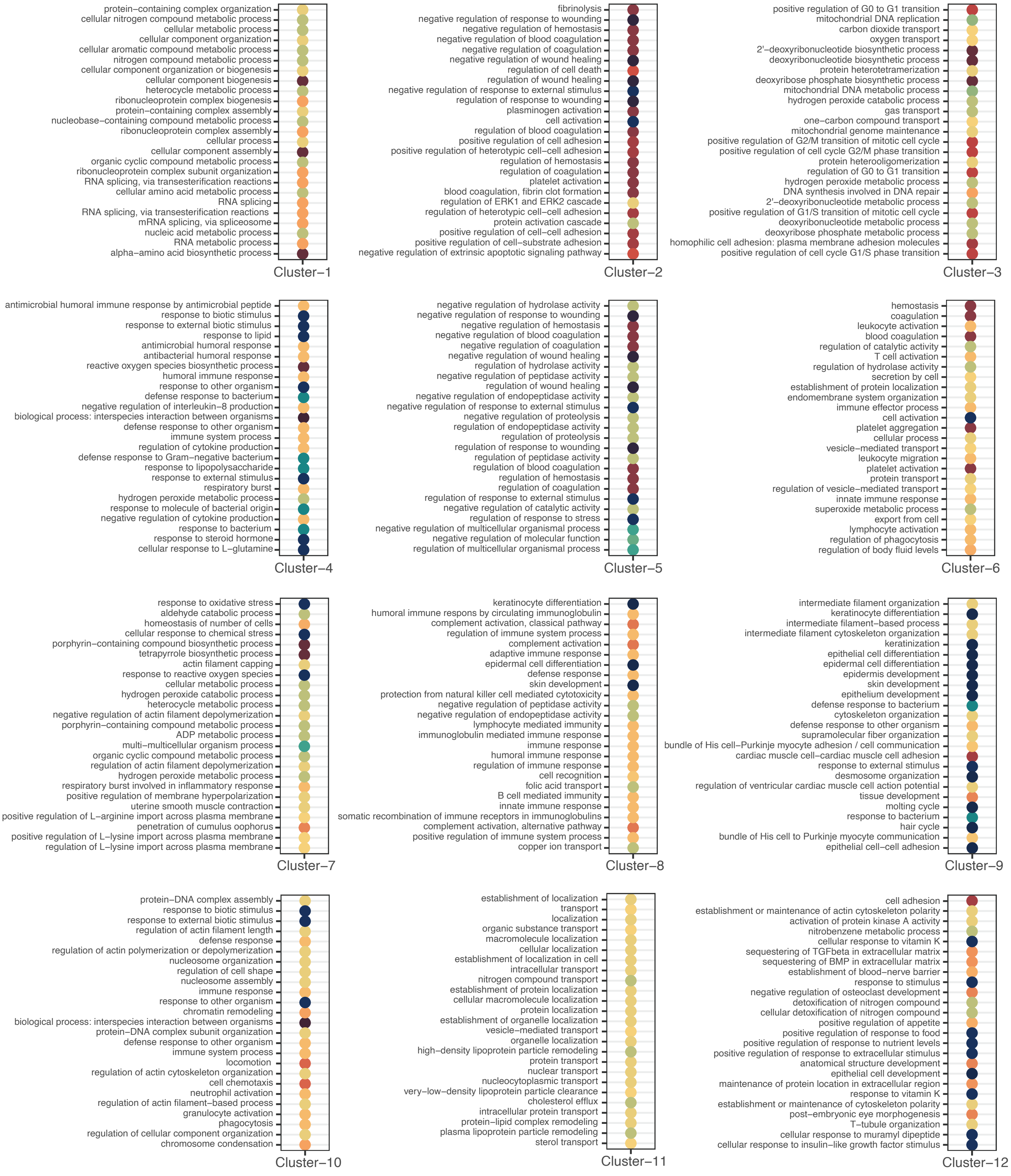


**Supplemental Figure 8 (page 1/2)**

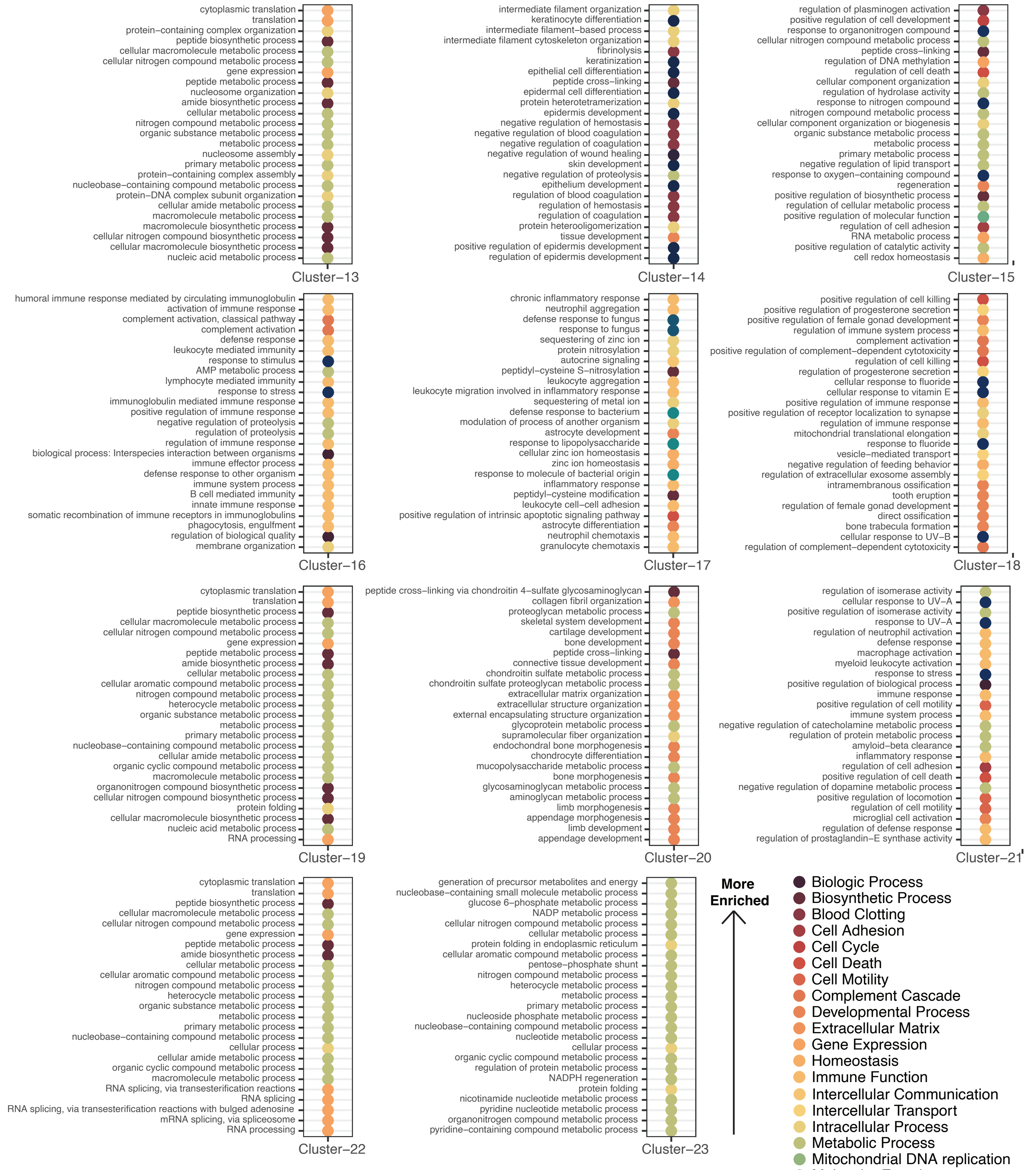


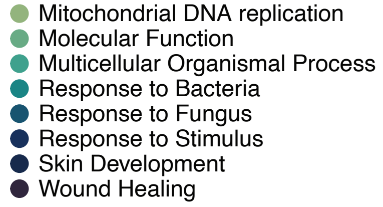


**Supplemental Figure 8 (page 2/2): Enriched GO biologic processes for each of the 23 k-means protein clusters.** To determine the key biologic processes associated with each protein k-means cluster, proteins within each cluster were submitted as unranked lists to the GO Enrichment analysis tool for evaluation with the PANTHER Overrepresentation test. Details for this analysis are included in supplemental table 8. This figure displays the top 25 most enriched GO biologic process each of the 23 k-means protein clusters. For each biologic processes are ordered from most significantly enriched (smallest FDR q-value) at the top to least enriched (largest FDR q-value) at the bottom. Color of the point indicates the broader biologic classification.
