# Supplementary material for: What is slough? Defining the proteomic and microbial composition of slough and its implications for wound healing
Source: Wound Repair Regen. 2024 Apr 1;32(6):783–98. doi: 10.1111/wrr.13170 (PMC11442687; doi:10.1111/wrr.13170)
Supplement: Supplementary file 1 — FIGURE S1. Photos of subject wounds before debridement procedure. [file WRR-32-783-s002.pdf]

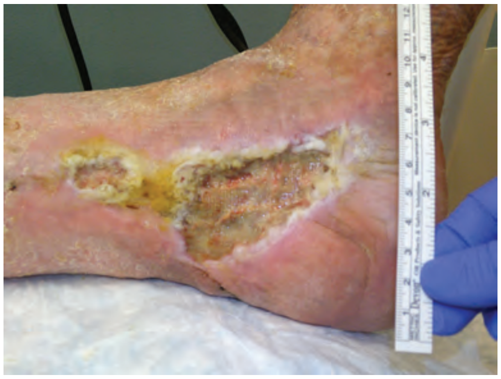

Subject -001

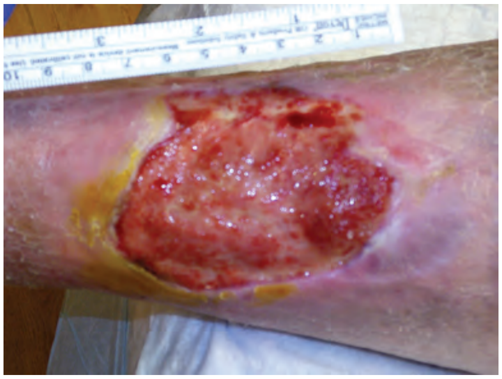

Subject -002

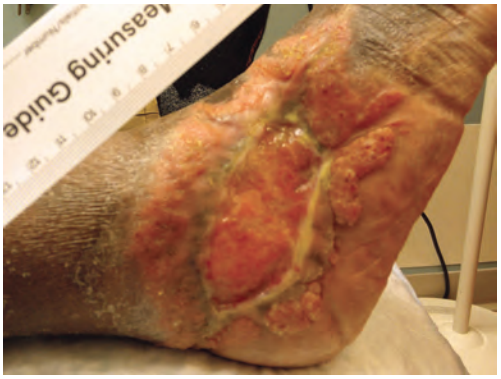

Subject -003

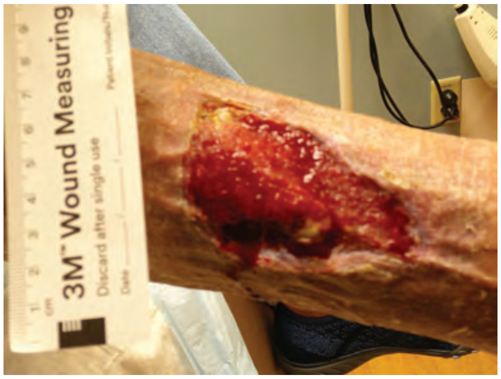

Subject -004

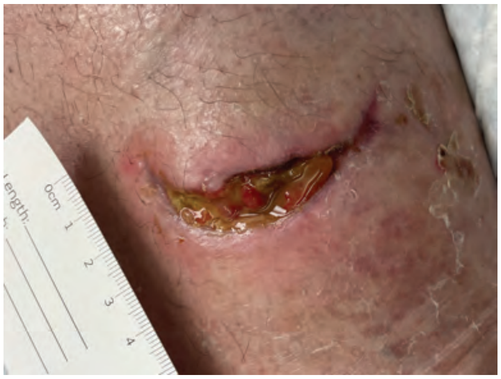

Subject -005

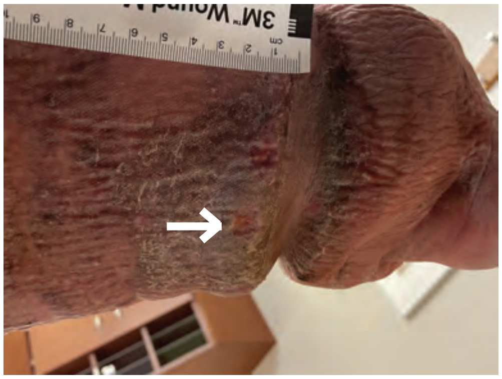

Subject -006

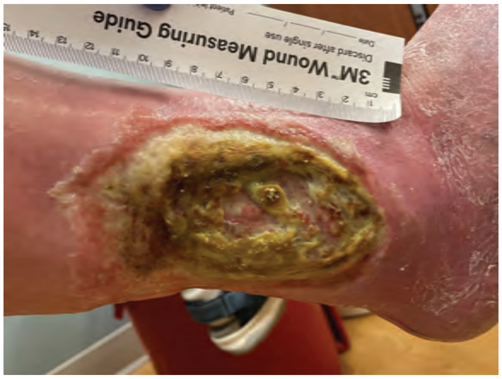

Subject -007

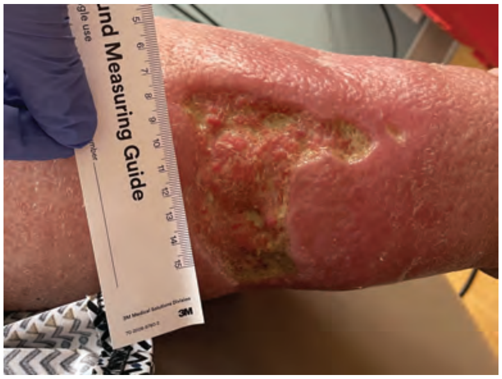

Subject -008

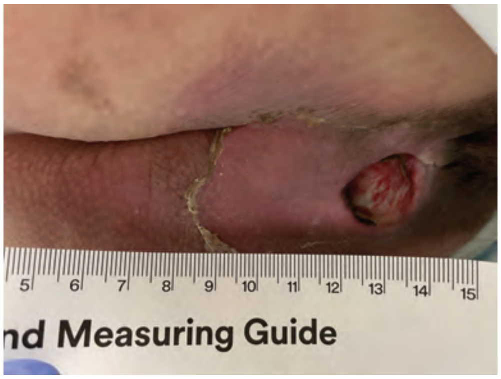

Subject -009

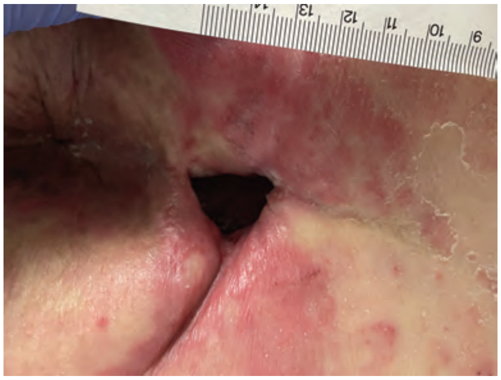

Subject -010

Supplemental Figure 1: Photos of subject wounds before debridement procedure.
